# Real-world walking speed as a digital biomarker and outcome measure for clinical trials—a systematic review, regulatory status and future directions

**DOI:** 10.3389/fdgth.2026.1726549

**Published:** 2026-02-17

**Authors:** Margaux Poleur, Cyril Tychon, Stephen Gilbert, Martin Daumer, Laurent Servais

**Affiliations:** 1 University Department of Neurology, Citadelle Hospital, Liège, Belgium; 2 Department of Pediatrics, University of Liège, Liège, Belgium; 3 Else Kröner Fresenius Center for Digital Health, TUD Dresden University of Technology, Dresden, Germany; 4 Faculty of Business and Economics, TUD Dresden University of Technology, Dresden, Germany; 5 SLCMSR E.V. - The Human Motion Institute, Munich, Germany; 6 Department of Paediatrics, MDUK Oxford Neuromuscular Centre, University of Oxford, Oxford, United Kingdom

**Keywords:** digital health, outcome measure, real-world, regulatory approval, walking speed

## Abstract

**Introduction:**

Walking speed is a key measure of health and mobility across a wide range of diseases. Traditional gait assessments in clinical settings may not accurately reflect real-world mobility patterns. Wearable sensors offer an ecologically valid alternative by capturing every movement in daily life, but there are few robust, validated reports. We aimed to identify evidence on real-world gait speed measurements that have received or are seeking regulatory approval from agencies such as the European Medicines Agency and the U.S. Food and Drug Administration.

**Method:**

We conducted a systematic review following a comprehensive search strategy using the Ovid platform, guided by pre-defined selection criteria and in accordance with the Preferred Reporting Items for Systematic Reviews and Meta-Analyses statement. We also manually searched the websites of key regulatory agencies and the ClinicalTrials.gov database.

**Results:**

Our search identified 503 records, of which 10 met the inclusion criteria. Most studies were part of large-scale initiatives, including the qualification of the Stride Velocity 95th Centile and the MOBILISE-D project. No device or outcome measure that assesses walking speed in real-world conditions has been fully validated by the FDA. We found four letters of intent on the FDA website related to this concept. One outcome, the 95th centile of stride velocity, has been approved by the EMA as a primary endpoint for assessing ambulant patients with Duchenne Muscular Dystrophy.

**Conclusion:**

Despite the potential of wearable devices to enhance drug development and clinical decision-making, real-world walking speed remains insufficiently validated across most conditions because data is missing. The widespread adoption of digital outcomes to assess ambulation will require extensive validation efforts, regulatory pathway adaptations, and improved standardization of devices, algorithms, and study methodologies.

**Systematic Review Registration:**

https://www.crd.york.ac.uk/PROSPERO/view/CRD42025633578, PROSPERO CRD42025633578.

## Introduction

Walking ability is a key determinant of quality of life as it is essential for many activities of daily living (1) and to maintain independence (2). Because walking relies on multiple physiological systems, gait assessment has become a central component of clinical evaluation in neurological (3, 4), cardiovascular, pulmonary (5) diseases.

It can be characterized by qualitative, functional gait parameters (i.e., speed, stride length) and quantitative, activity-based gait parameters (i.e., stride per hour, walking distance). Among these parameters, walking speed has been associated with disease severity, prognosis, and functional status across numerous diseases including multiple sclerosis (MS), heart failure, and stroke (6–8). Also in healthy elderly patients, slower walking speed could predict a higher risk of mortality (9, 10) and dementia (11). Walking speed has been extensively utilized as a meaningful outcome measure in clinical trials, particularly within neurological research (12, 13).

Walking tests are currently conducted in controlled environments and therefore susceptible to contextual biases. Among them, the Hawthorne effect, defined as a change in behavior as a response to observation and assessment (42). This awareness may lead participants to intentionally or unintentionally alter their performance. For example, some individuals might walk faster than usual to appear more capable, while others might deliberately walk more slowly if they know that reduced walking speed increases their eligibility for an appealing clinical trial. Moreover, such tests provide only a limited snapshot of a patient's walking ability, potentially influenced by transient factors like mood, fatigue, or stress. These biases potentially result in false-positive or false-negative trial outcomes.

Although real-world monitoring with wearable sensors may initially influence patient behavior due to awareness of being monitored, an effect that is deliberately leveraged by many commercial activity trackers to encourage increased step-based performance, continuous data collection over extended periods (e.g., several weeks to months) substantially mitigates this influence. Over time, initial behavioral modifications are expected to stabilize into habitual patterns that more accurately reflect the patient's true functional performance in daily life, thereby providing a reliable measure of real-world walking speed that is robust to transient behavioral changes. Despite this promise, most diseases still lack consensus on which digital mobility variables best capture the clinical picture and sensitively detect meaningful change. Recent reviews highlight many small initiatives in various fields but only few reports of robust validated outcomes (14–16). These reviews describe a wide range of commonly used sensor modalities, including inertial measurement units (IMUs) composed of accelerometers and gyroscopes, pressure sensors, surface electromyography, and instrumented insoles, as well as their diverse body placements. Typical sensor locations include the wrist, ankle or foot, and lumbar region, selected according to the functional domain and disease being monitored. These sensors have shown promise in detecting subtle changes (16) or fluctuations (14) in motor performance with greater sensitivity than conventional clinical assessments. Previously mentioned reviews emphasized the need for further validation of digital outcomes, yet none identifies outcomes or devices that have received regulatory approval or undergone any form of qualification.

Regulatory qualification is a critical step to integrate digital outcomes into evidence-based medicine. It ensures their acceptability for use in clinical trials and healthcare decision-making (17). Without regulatory qualification, digital outcomes may be considered exploratory and not suitable as primary or secondary endpoints in regulatory trials. This limits their use in drug development, labeling claims, and ultimately their impact on patient care. Moreover, the qualification process promotes standardization, facilitates study comparisons, and integrates findings into both clinical practice and research.

This review synthesizes current knowledge on the use of real-world walking speed as an outcome measure for patient evaluation. It also emphasizes digital outcomes and wearable health technologies that have or seek to achieve regulatory qualification from agencies such as the U.S. Food and Drug Administration (FDA) and the European Medicines Agency (EMA). Additionally, we aim to identify and discuss the main challenges in the translation of real-world walking speed metrics into regulatory-qualified clinical outcomes.

## Methods

### Data selection

First, we completed a systematic review. The search strategy is explained in our published protocol, available at the International Prospective Register of Systematic Reviews (Registration: CRD42025633578) and adhered to the Preferred Items for Systematic Reviews and Meta-Analyses statement (PRISMA) (18)^.^ The PRISMA checklist and the complete search strategy are shown in Supplementary File S1, S2, respectively.

To achieve our objective of identifying methods that are in the advanced stages of the regulatory approval process, we chose to focus on final peer-reviewed papers accessible through MEDLINE and EMBASE. Subsequently, we conducted a review of the literature using a comprehensive search with pre-defined selection criteria and search strategy.

This approach allows us to systematically review the most current, high-quality evidence, particularly those studies that represent advanced stages of development—whether through late-phase clinical trials or post-marketing studies. We searched MEDLINE via Ovid on August 1, 2024, and EMBASE on December 15, 2025. The MEDLINE search was updated on the same day. We included English-language studies published from 2019 onward. As English is the predominant language of regulatory and scientific communication, relevant evidence intended to support regulatory approval is expected to be available in English. The search was limited to the most recent years to reflect the current regulatory landscape and contemporary evidentiary standards. In medical device development, regulatory efforts that have not progressed within this timeframe are unlikely to succeed without substantial new evidence or technological modification; consequently, older unsupported studies have limited relevance for assessing present-day regulatory feasibility.

### Eligibility criteria

Eligibility of records was assessed using the PICO framework to define inclusion and exclusion criteria based on the four key components: Population, Intervention, Comparison, and Outcome, and the additional criteria of Type of Study, Language of Publication, and Publication Date (Supplementary File S3).

### Study selection and data extraction

After running the search strategy, citations of all the selected papers were uploaded into Covidence, a software platform designed to conduct systematic reviews. Duplicates were manually eliminated. Margaux Poleur (MP) and Cyril Tychon (CT) independently screened titles and abstracts and subsequently full texts for eligibility. Disagreements were resolved by a third reviewer, Laurent Servais (LS). We included reports that assessed real-life walking speed in humans. After this initial screening, the full texts of all remaining reports were examined in a second round of review. During this stage, studies reporting proof-of-concept or pilot data were excluded unless they could be clearly linked to a broader body of evidence supporting a given outcome or device. Such evidence could consist either of external supporting material, including information available in regulatory sources (e.g., qualification reports, regulatory submissions), or of multiple related studies which, when considered together, constituted a sufficiently large and coherent body of evidence. No restriction was applied regarding gender, age or ethnicity. The two reviewers compared their findings, and potential disagreements were resolved by consensus or with the help of a third reviewer (LS).

Device and outcome characteristics were extracted from all data collected (outcome, targeted population/context of use, broader initiative, technology, number and location of sensors). Two assessors (MP and CT) extracted these data in Covidence. Results from both extractions were compared until assessors reached consensus.

The main outcomes reported in these studies, including validity, reliability, feasibility, accuracy, and sensitivity to change, were also extracted.

We define these categories as follows (19):

Feasibility evaluates how practical and acceptable a measurement tool is for use in real-world settings. This includes considerations such as ease of use, participant burden, data completeness, cost and compliance.
Reliability refers to the consistency and stability of a measurement under identical conditions.
Accuracy describes how close a measurement is to the preferred or accepted reference value. In digital outcomes, this typically refers to how precisely the device or algorithm measures the targeted parameter (e.g., actual walking speed) compared to precision measure (3D motion capture).
Validity refers to which extent a measurement tool accurately records what it is intended to measure. We extract known-group validity measure (ability to differentiate between controls and patient or patient with different level of disability or patient with different conditions) and construct validity [correlation with gold standard outcome, i.e., 6-minute walk test (6MWT) in DMD].
Sensitivity to change, or responsiveness, indicates a measurement tool's ability to detect slight but meaningful changes over time, especially in response to interventions or disease progression.
Minimal Clinically Important Difference (MCID) represents the smallest change in a measurement that is perceived as beneficial or meaningful by patients or clinicians. It helps determine whether observed changes are not only statistically significant but also clinically relevant.

### Additional data

We collected additional data to ensure that no crucial information was overlooked by our systematic methodology. As a second approach, to find research results that have regulatory visibility, we searched the websites of regulatory agencies. We focused on the U.S. Food and Drug Administration (FDA) and the European Medicines Agency (EMA) as these regulatory agencies are the most influential worldwide. Their expertise in the approval and regulation of medicines and medical devices make them well-positioned to provide an overview of the cutting-edge biomarkers. We considered initiatives that have demonstrated an intention to seek formal approval, as evidenced by the submission of a Letter of Intent (LOI) −a formal document submitted to a regulatory authority to communicate the intent to engage in a regulatory process (e.g., for drug or device development).

We also directly contacted members of these agencies to collect additional relevant evidence. Based on the findings of the literature research we searched Medline and Clinicaltrial.gov database on December 1st, 2024, for ongoing studies linked to the identified and selected reports.

### Data analysis and synthesis

Data were synthesized using a narrative approach, given the heterogeneity in study designs, populations, and outcomes across the included papers. To facilitate comparison and highlight condition-specific findings, studies were grouped according to the disease or disorder they addressed. Within each group, we examined common themes, key outcomes, and methodological characteristics, providing a structured summary of the evidence for each condition.

### Quality assessment

We performed risk of bias assessment of the included studies, using the Mixed Methods Appraisal Tool (MMAT) for research papers or the Joanna Briggs Institute (JBI) critical appraisal tools for reviews. MP and CT independently assessed the quality of the included studies. Disagreements at any stage were resolved by discussion.

## Results

### Source of the data

#### Regulatory agencies web site and contact

There is no outcome or device that has been validated by the U.S. Food and Drug Administration (FDA) for the assessment of walking speed in real-life. We found 4 letters of intent on the FDA website (20) regarding our concept of interest. One of them applies for the use of Biostamp® in Huntington disease and it was declined by the FDA in 2020. Two LOI were accepted regarding the use of actibelt® in MS and sarcopenia respectively. The use of Actimyo® in Duchenne Muscular Dystrophy was proposed and accepted in the most recent LOI.

Only one outcome or device was identified as regulatory approved in the EU. The EMA website published the complete qualification package (21) of the stride Velocity 95th centile (SV95C) as primary endpoint in superiority studies for ambulant patients with DMD above 4 years old. (22, 23).

There is at least one device in the regulatory pathway in the EU with a focus on real-world walking speed. The FDA website published a briefing (24) that asks for EMA scientific advice on the usage of changes of real-world walking speed as measured with actibelt technology as phase III endpoint for MS, recovery after surgical treatment of hip fracture and sarcopenia. The EMA's unpublished scientific advice, which included the key questions to be addressed, was shared with the applicants in 2017, prior to the publication of the €50 million IMI call (25).

#### Literature review

**Figure 1:**
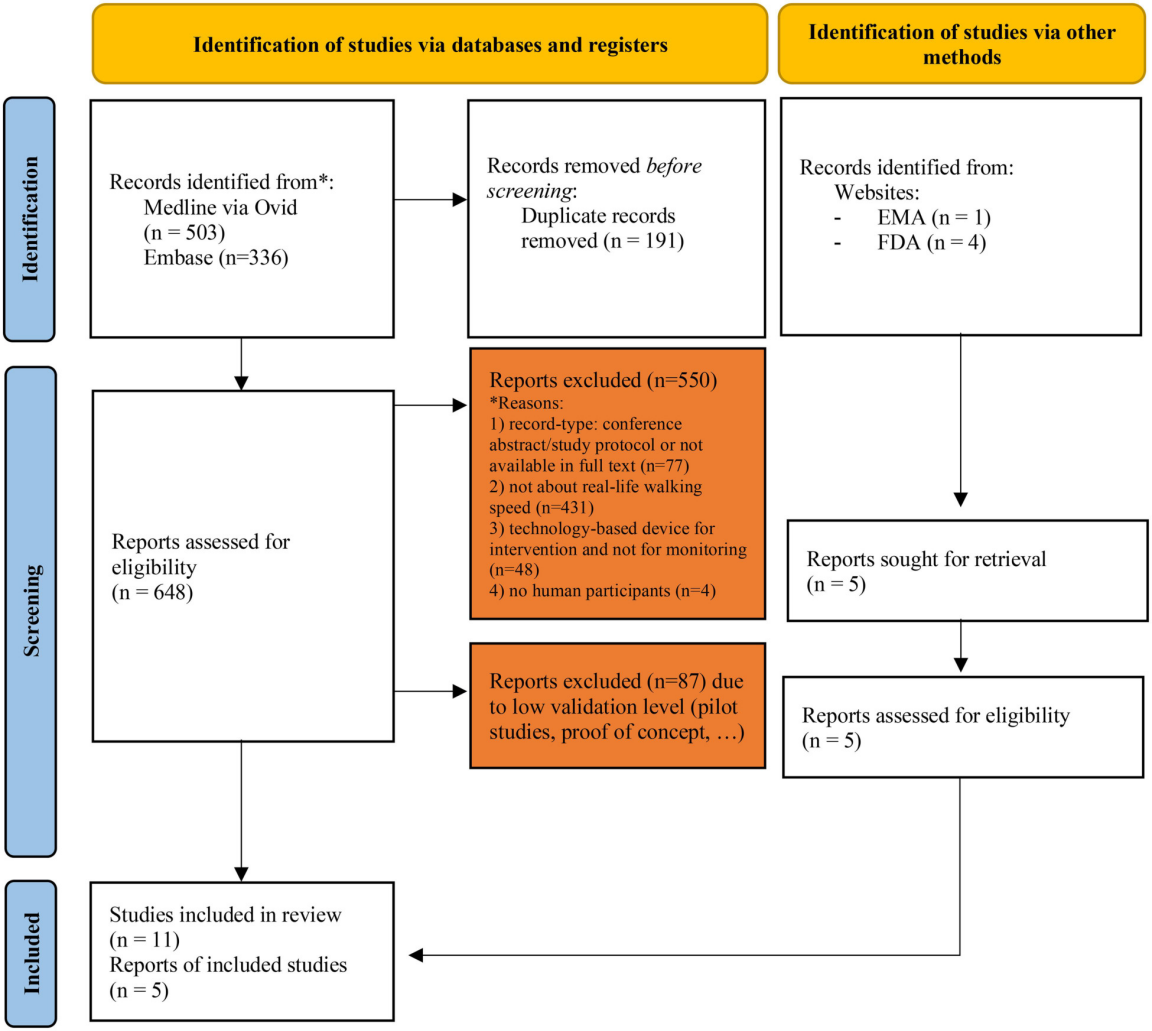
Flow-chart of selection process. This work is licensed under CC BY 4.0. To view a copy of this license, visit https://creativecommons.org/licenses/by/4.0/. source: page MJ, et al. BMJ 2021;372:n71. doi: 10.1136/bmj.n71.

A flow-chart of the process used for inclusion of clinical studies in our analysis is shown in Figure 1. Our literature search identified 648 records. Of these, 550 were excluded as they did not meet the scope of the review. An additional 87 studies were excluded because they consisted of small pilot studies or proof-of-concept reports without sufficient relevance for validation purposes. Information on collected data are summarized in Tables 1, 2. All papers except one were part of broad initiatives, namely the qualification of the SV95C and MOBILISE-D. One MOBILISE-D publication was a perspective article describing the rationale, regulatory context, and anticipated impact of digital endpoints but did not report original empirical data and was therefore not included in the synthesis of results (43). The last paper is linked to the LOI submit to the FDA for the use of actibelt® in sarcopenia.

**Table 1:** Characteristics of included studies.

Broader initiative	Author and year of publication	Disease	Number, age, sex of participants [F/M, age ± SD (range)]	Device name	Technology used	Outcome	Number/location of sensor(s)	Context of use
In collaboration with MOBILISED-D	Kirk. et al. (26)	Parkinson disease	88 PD, 111 controls	AX3 (Axivity, United Kingdom)	Tri-axial accelerometer	Real-world walking speed (RWS)	1 sensor (lower back)	Participants underwent clinical and real-world assessments at 18, 36, 54, and 72 months following baseline assessment. Participants wore the device for 7 days
MOBILISED-D	Kirk et al. (47)	Congestive heart failure (CHF), chronic obstructive pulmonary disease (COPD), healthy adult (HA), multiple sclerosis (MS), Parkinson's disease (PD) and proximal femoral fracture (PFF)	97 participants: 17 controls (72.35 ± 6.00 y.o), 9 chronic heart failure (68.00 ± 12.80 y.o), 17 chronic obstructive pulmonary disease (69.35 ± 9.10 y.o), 13 multiple sclerosis (47.23 ± 11.09 y.o), 15 Parkinson's disease (69.20 ± 7.48 y.o), 11 proximal femoral fracture PFF (79.70 ± 6.86 y.o)	Dynaport MM + (McRoberts)	Tri-axial accelerometer, triaxial gyroscope	Real-world walking speed (RWS)	1 sensor (lower back)	Participants were monitored in the laboratory (INDIP reference system) and the real-world (2.5 h). Participants were also asked to wear Dynaport and a multisensor INDIP reference system
MOBILISED-D	Boehme et al. (43)	Frailty, heart and lung diseases, neurologic and neurodegenerative diseases, cancer	N/A	N/A	Wearables + smartphones application	Real-world walking speed (RWS)		
MOBILISED-D	Buekers et al. (48)	chronic obstructive pulmonary disease (COPD), multiple sclerosis (MS), Parkinson's disease (PD) and proximal femoral fracture (PFF)	565 chronic obstructive pulmonary disease (206/509, 68 ± 8 y.o), 558 multiple sclerosis (358/200, 52 ± 11 y.o), 543 Parkinson's disease (197/346, 66 ± 10 y.o), 487 proximal femoral fracture PFF (319/168, 77 ± 10 y.o)	Move-Monitor + (McRoberts) or AX6 (Axivity, United Kingdom).	Wearable device	Multiple gait and activity outcomes, including walking speed	1 sensor (lower back)	Participants were asked to wear a single wearable, 24 h/day for seven days:
SV95C qualification	Lilien et al. (44)	Duchenne Muscular Dystrophy (DMD), type 3 spinal muscular atrophy (SMA)	23 ambulant	ActiMyo (Sysnav, France).	Tri-axial accelerometer, gyroscope, magnetometer and barometer	Digital 6MWD	1 sensor (lower back)	Participants performed a 6MWT wearing the device. Some patients performed a second test 6 months later
SV95C qualification	Poleur et al. (27)	Healthy subjects	91 controls (41/50, 16.9 ± 16 y.o)	ActiMyo (Sysnav, France).	Tri-axial accelerometer, gyroscope, magnetometer and barometer	Stride length and stride velocity were studied as the medians (SL50C and SV50C) or the 95th centiles (SL95C or SV95C)	2 sensors (dominant wrist and ankle)	The sensors was worn for 1 month at baseline and at 12 months
SV95C qualification	Gidaro et al. (46)	Limb girdle muscular dystrophy type R2 (LGMDR2) and facioscapulohumeral muscular dystrophy (FSHD)	18 participants: 8 FSHD, 10 LGMDR2	ActiMyo (Sysnav, France).	Tri-axial accelerometer, gyroscope, magnetometer and barometer	Mean and 95th percentile of stride length (SL95C), Mean and 95th percentile of stride velocity (SV95C), total number of stride, walked distance	2 sensors (dominant wrist and ankle)	Patients presented here were included in ATYR1940-C-004 study (NCT02579239), in which digital outcomes were included as exploratory endpoints. The ATYR1940-C-004 study was a phase 1b/2, multi-center, open-label, intrapatient dose-escalation study evaluating the safety, tolerability, immunogenicity, and biological activity of ATYR1940, a histidine aminoacyl tRNA synthetase promoting skeletal muscle homeostasis and resetting the immune system to control or reduce tissue damage. They wore the DHT continuously from screening to the end of the trial (4 months)
SV95C qualification	Servais et al. (22)	Duchenne Muscular Dystrophy (DMD)	Unknown	Syde (Sysnav, France).	Tri-axial accelerometer, gyroscope, magnetometer and barometer	Stride velocity 95th centile (SV95C)	2 sensors (wrist and ankle)	DMD patient were recorded during 1 month at baseline and at 6 month
SV95C qualification	Servais et al. (45)	Duchenne Muscular Dystrophy (DMD)	Various DMD cohorts	ActiMyo (Sysnav, France).	Tri-axial accelerometer, gyroscope, magnetometer and barometer	Stride velocity 95th centile (SV95C)	2 sensors (dominant wrist and ankle)	To assess accuracy, the 6-min walk test was assessed simultaneously using the wearable device/system and the classical method for 31 tests performed by 23 patients in a broad range of clinical conditions. To assess reliability, they studied the relationship between the recording period averaging and the variability of the measure by tracing the variance in 28 patients tested in a non-controlled setting. The relationship between stride velocity, stride length, and distance per hour and the 6-min walk test, North Star Ambulatory Assessment score, and 4-stair climbing test score was assessed in 45 DMD patients. The sensitivity to change was assessed on 6 month in 31 patients and on a subgroup of 20 patients older than 6 years and walking <450 m in the 6-min walk test.
SV95C qualification	Haberkamp et al. (28)	Duchenne Muscular Dystrophy (DMD)	Boys [8.3 ± 2.1 (5–14) y.o]	ActiMyo (Sysnav, France).	Tri-axial accelerometer, gyroscope, magnetometer and barometer	The 95th centile of the stride velocity (SV95C), the median stride velocity, the 95th centile of the stride length the median stride length and the distance walked/recorded hour	2 sensors	Home-based environment, 1-month recording
FDA Letter of intent regarding the use of actibelt in Sarcopenia	Mueller et al. (29)	Sarcopenia	26 slow-walking elderly subjects (15/11) 217 patients with sarcopenia (126/91, 79 ± 5.45 y.o)	actibelt RCT2 (Trium Analysis Online, Germany)	Tri-axial accelerometer	Multiple gait parameters	1 sensor (waist)	During the validation study, the subjects completed a parcours course, at least twice, through the clinic that was designed to simulate a real-world environment. Patients were monitored over 25 weeks during a phase 2b interventional clinical trials, both during and between visits. Patients were instructed to wear the device continuously for a minimum period of 5 days before each planned clinical site visit.

**Table 2:** Psychometric properties and validation evidence for real-world walking speed measures.

Broader initiative	Author and year of publication	Feasibility/Compliance	Accuracy	Reliability	Clinical validity	Sensitivity to change	MCID	Other
MOBILISED-D	Kirk. et al. (26)				RWS was significantly different between PD and controls at each time point and walking bout. At 36-month, there was no significant association with MDS-UPDRS III score with RWS at all WBs. There was a significant negative association with the MDS-UPDRS III at WBs between 30 and 60 s (*p* = 0.047). There was no association between change in RWS with changes in MDS-UPDRS III at all WBs and each other WB duration threshold.	RWS significantly slowed in PD by 2 cm/s per year (*p* = 0.014) and in controls by 1 cm/s per year (*p* = <0.001), when aggregated within WBs >10 s. RWS calculated within each WB threshold slowed significantly at each WB duration, excluding long WBs (>60 s) in PD. Rate of decline in RWS was larger in PD in comparison to controls, at each WB duration threshold, excluding >60s where we observed no difference with controls.		
MOBILISED-D	Kirk et al. (47)		In controlled environment, the mean absolute error (MAE) and mean relative error (MRE) for walking speed estimation ranged from 0.06 to 0.12 m/s and−2.1 to 14.4%. Real-world MAE ranged from 0.09 to 0.13, MARE from 1.3 to 22.7%. Lower errors were observed for cohorts without major gait impairments, less complex tasks, and longer walking bouts. ICC ranged from 0.79 to 0.91 in controlled environment and 0.57 to 0.88 in unsupervised environment					
MOBILISED-D	Boehme et al. (43)							
MOBILISED-D	Buekers et al. (48)	The required minimum daily wear-time ranged from no requirement (13% of parameter–condition combinations) to more than 14 h (19%), with longer wear times generally required for walking activity than for gait parameters. The minimum number of monitoring days varied from 1 day (17%) to more than 7 days (6%), with higher requirements observed for parameters that have not yet been clinically validated. No evidence was found for an effect of weekends or health condition on parameter reliability.						
SV95C qualification	Lilien et al. (44)	In 2012, the device was used in a clinical study for controlled and home-based monitoring of upper-limb movements in non-ambulant DMD patients (NCT01611597) which demonstrated the autonomy and feasibility of device use. Patient compliance tends to decrease over time.	The difference between the distance measured by the inertial device and the reference 6 MWT (after correction for the length of the turn around the cones) was within 5%		The validated 6 MWT and the North Star Ambulatory Assessment (NSAA) have been correlated with the device's variables.			
SV95C qualification	Poleur M., et al. (27)	Among the 91 healthy controls who wore the wearable device for one month, 90% recorded more than 50 h, and 77% recorded more than 180 h at baseline. During the second period, 86% recorded more than 50 h, and 73% recorded more than 180 h. The main reason reported y subjects who recorded less than 50 h was “technical issues”.			In comparison to ambulant DMD patients of the same age, healthy children present a 67.3% higher SV95C. The difference for other variables was not as extreme, and there is some overlap between DMD and control participants (SL50C 31.39%, SL95C 52.41%, SV50C 25.72%, distance walked/hour 63.13%) 6MWD was significantly correlated with SV50C, SV95C, and SL95C in the younger group. In the adult group, 6MWD was only correlated with “maximum performance” measures SV95C and SL95C. Among the group aged 5 to 17 years, height was highly correlated with age, 6MWD, 10MW, SV50C, SL50C, and weakly correlated with SV95C and SL95C. In adults, height was only correlated with stride length measures SL50C and SL95C.	In the younger group, only SL50C increased after one year. We did not observe a significant change after a year in adults in stride length, stride velocity.		
SV95C qualification	Gidaro, et al. (46)	18 patients enrolled in the study, 10 were ambulant and compliant for wearable sensors analysis, 3 patients were not sufficiently compliant (less than 22 days of data), 3 patients were non-ambulatory, 2 patients were not given the sensor.		All the measurements showed high reliability according to ICC values (all >0.9). Median and 95th percentile of stride length (SL95C), Median and 95th percentile of stride velocity (SV95C) showed lower SEMs (all <0.03) compared to Total number of stride and walked distance (40.2 and 28.5).	Median speed, length, SV95C and SL95C were significantly different at baseline between subjects with FSHD and LGMDR2. Median speed (R = 0.842), median length (R = 0.866), SV95C (R = 0.866) and SL95C (R = 0.915) strongly correlate with Manual muscle testing.	Slight decreases in all variables were observed in subjects over time with significant decrease (*p* ≤ .05) for median speed, SV95C and SL95C.		
SV95C qualification	Servais et al. (22)	A total of 180 h was optimal, with good compliance and low measurement variability; 90% of patients recorded 180 h over 1 month.	ActiMyo stride detection is derived from the 3D trajectory of the ankle, providing a 98% stride detection success rate over 2,000 atypical strides, including small or side step. In healthy volunteers, ambulation parameters were captured with similar levels of accuracy to motion capture, with a peak-to-peak discrepancy rate below 2.5%		SV95C had moderate but significant correlations with the 6MWT and NSAA	After 6 months, in patients aged 7 years and above with baseline 6MWT below 450 m, stride length 95th centile declined by 2.4% (*p* < 0.05), median stride velocity by 4.7% (*p* < 0.05), and SV95C by 8.5% (*p* < 0.01. SV95C is sensitive to the positive functional changes associated with steroid treatment; in steroid-naïve patients, steroid initiation resulted in an 11.6% increase in SV95C after 6 months, while age-matched patients receiving stable steroid treatment experienced a 6.8% decline.	The MCID for SV95C was calculated using the distribution-based methods described by McDonald et al. as 0.1 m/s (a relative 6.24% decline), corresponding to a 36 m difference in 6MWT (30 m is the accepted 6MWT MCID in DMD trials).	Large sample sizes are required for studies employing the 6MWT as a primary endpoint. In the phase III ataluren study for DMD, an estimated 105 patients per treatment arm were needed to show a 30 m difference in 6MWT at 48 weeks with 85% power [31]. For SV95C, 14 patients per arm with DMD aged 7 years or above (with 6MWT baseline < 450 m) provides sufficient statistical power (80%) to demonstrate significant stabilization of motor function with treatment at 6 months
SV95C qualification	Servais et al. (45)		The difference between the distance measured during the 6-min walk test using the wearable device/syste and the corrected reference was within 5%.	They showed a good stability with low variability from 2.22 up to 4.41% for the 95th percentile of stride length and 95th percentile of stride velocity, respectively, for 180 h of wearable device and system use.	Analysis showed a good correlation of 95th percentile of stride length and 95th percentile of stride velocity with the baseline 6-min walk test (0.68 and 0.54, respectively) and with the North Star Ambulatory Assessment (0.78 and 0.64, respectively).	They observed a significant decline in 95th percentile of stride length (2.4%), median stride velocity (4.7%), and 95th percentile of velocity (8.5%) over 6 months for patients older than 6 with a baseline 6-min walk distance below 450 m.		
SV95C qualification	Haberkamp et al. (28)	The SV95C was calculated from a period of 180 h of summed recorded data per individual. This period seemed to ensure low variability while keeping good compliance. Daily recording including weekends is needed for representative data. Therefore, in ambulant DMD patients, 30 day wearing periods are proposed to ensure that sufficient data are generated during a set period for almost all patients.	The distance measured from reconstruction of foot trajectory of ambulant patients as assessed by the magneto-inertial sensor corresponds to the real distance as measured manually. Accuracy of measurements using the device compared to an optical motion capture system in healthy subjects.		SV95C was significantly correlated with the validated 6 MWT and NSAA. Baseline normative data indicate that stride speed and stride length clearly discriminate between controls and DMD, especially when expressed as the 95th centile.	Longitudinal (31 patients at 6 months and 11 patients at 12 months for SV95C vs. 6 MWT) changes observed for the SV95C are in line with those observed in DMD studies for 6 MWT.	MCID of 0.1 m/s for SV95C	
FDA Letter of intent regarding the use of actibelt in Sarcopenia	Mueller et al. (29)	Ultimately, 9,668 patient-days of accelerometry data were collected from 192 patients and 32 sites. This demonstrates the feasibility of continuous monitoring in global clinical trial settings and elderly populations. We observed that patient compliance patterns were highly variable, with some patients greatly exceeding the requested wear-time and others contributing far less	In-clinic measurement of gait speed in frail slow-walking adults with a residual standard error of 0.08 m per second in the independent validation study and 0.08, 0.09, and 0.07 m per second for the 4 m walk test (4mWT), 6-min walk test (6MWT), and 400 m walk test (400mWT), respectively.		They found positive associations between the short 4mWT gait speed assessment and gait speed in bouts between 5 and 20 steps (correlation of 0.23) and longer 6MWT and 400mWT assessments with bouts of 80 to 640.			

#### Clinical trial database

We identified one and 14 ongoing studies related to the MOBILISE-D initiative and SV95C qualification, respectively.

### Data by disease

#### Muscular dystrophies

In 2023, the EMA qualified the first digital outcome for the use as primary endpoint in clinical trials for Duchenne Muscular Dystrophy (DMD). This outcome, the Stride Velocity 95th centile (SV95C), represents the 5% fastest strides in daily living, measured with an inertial wearable device called Actimyo®. This achievement relies on its excellent metric properties (21). Accuracy was demonstrated in comparison with a motion capture gold standard in controls and by comparison with physiotherapist evaluation during a 6MWT in DMD patients (44). SV95C significantly correlated (*p* < 0.001) with the 6MWD, North Star Ambulatory Assessment (NSAA) and the timed 4-stairs climb (4SC) at baseline and every 3 months during a 1-year follow-up (45). The intraclass correlation coefficient (ICC) calculated on 2 successive 1-month recording periods in 52 patients with DMD was 0.97 establishing an excellent reliability. SV95C could differentiate between patients and age-matched controls as well as DMD from different age groups. The sensitivity to detect negative change was demonstrated by statistically significant decline of the SV95C at each time point (*p* < 0.001) as early as 3 months (*n* = 81).

These results have been replicated on independent populations of patients (30) and compared to large normative database (27).

SV95C has already been used as secondary endpoint in phase 3 clinical trials (NCT05096221, NCT06138639, NCT04906460, NCT05524883, NCT03039686). Results from one of them has been published (NCT03039686) and confirms the good metric properties of SV95C. An ongoing multicentric longitudinal study (NCT05982119) is recruiting controls and patients living with DMD for longer than 2 years old as well as participants with other neuromuscular diseases including Facio-scapulo-humeral dysplasia (FSHD).

A longitudinal pilot study (*n* = 18) using SV95C among others enrolled patients living with FSHD (46). This study showed its good reliability (ICC > 0.9), external validity as well as a significant decline over a 4 months-period.

#### Parkinson disease (PD)

We found no fully regulatory-qualified outcomes related to real-walking speed (RWS) in Parkinson's disease. However, we included two studies that is part of the MOBILISE-D initiative, and another conducted in collaboration with it. The latter, a 72-month study using AX3®, included 88 patients living with PD and 111 controls to assess real-world walking speed based on a 7-day recording period (26). RWS was significantly different between PD and controls but did not correlate with MDS-UPDRS III score at walking bouts between 30 and 60 s. RWS more significantly declined in PD (−2 cm/s/year, *p* = 0.014) than in controls (−1 cm/s/year, *p* = <0.001). A separate study from the same initiative used the same outcome measure but a different device in patients with various conditions (congestive heart failure, chronic obstructive pulmonary disease, MS, Parkinson's disease, proximal femoral fracture and controls) (47). They showed on 15 patients living with PD that walking bout detection was accurate (0.92), but less sensitive (0.60) than laboratory conditions. The absolute error was 11 cm/s and 17 cm/s in laboratory and daily living conditions, respectively. A recent study shows that gait and walking activity measures reached acceptable reliability using the same threshold of >12 h of wear-time for at least 3 days (48). The ongoing MOBILISE-D extension study (NCT05874739) is an observational study aimed to investigate the validity of digital mobility outcomes quantified from a device-agnostic analytical pipeline on a larger cohort (up to 411 participants with PD and 240 age and gender-matched controls).

#### Sarcopenia

We identified an accepted LOI from 2019 on the FDA website for the use of a waist worn tri-axial accelerometer (actibelt®) to assess real-world walking speed. We identified one study that may serve as preliminary work for this LOI, as it utilizes the same device to record various gait parameters. The study enrolled 217 patients with sarcopenia and 26 controls in a 25-weeks study. Patient wore a tri-axial accelerometer on the waist for a minimum period of 5 days before each planned clinical site visit. Data were collected in 192 patients on 32 sites. The residual standard error for gait speed ranges from 7 to 9 cm/sec. The study found a weak to moderate relationship between short (4-minute) and long (6-minute and 400-meter) walking tests and daily living mean gait speed during short and long walking bouts, respectively (29). This study is based on a validation study evaluating the accuracy of the actibelt® speed algorithm, “step wave”, which concluded that the deviation was less than 1% in step detection and less than 0.05 m/s in gait speed measurements compared to the reference, even for slow-walking subjects (31).

#### Multiple sclerosis

To date, no outcome measures related to real-world walking speed in MS have achieved regulatory qualification. We identified another accepted LOI on the FDA website for the use of a waist worn tri-axial accelerometer (actibelt®) to assess real-world walking speed. A 2012 study (32) showed that in 51 patients living with MS, the actibelt® speed algorithm “SVM” overestimated the mean walking speed during a 6MWT compared to standard method. The absolute error ranged from 2 to 26 cm/s, with a strong linear association between the error and Expanded Disability Status Scale (EDSS) (*r* = 0.68, *p* = 0.0001) along with the average of actual and actibelt® walking speed (*r* = 0.71, *p* = 0.0001) in the overall sample. In association with the LOI, we identified an unpublished multicenter study involving 74 patients living with MS, which demonstrated that a 0.1 m/s decrease in mean daily living walking speed—measured with actibelt® over 7 consecutive days—approximately corresponded to a 2-point increase on the EDSS (33). Evidence from the Mobilise-D project indicates that reliable real-world digital mobility outcomes can be achieved in MS with more than 12 h of daily wear over at least 3 days, independent of weekend inclusion and consistent across levels of physical capacity (48).

#### Huntington disease (HD)

Our review did not identify any outcome measures that are fully qualified by regulatory authorities in HD. MC10, the company that developed Biostamp®, a body-worn wearable sensor, submitted a LOI to the FDA in 2020. They aim to develop an outcome measure focused on the specific gait abnormality in HD for early detection and assessment of the disease progression in clinical trials. This LOI was rejected by regulatory authorities because their aim was to measure gait parameters that lacked a clear functional impact. We did not find any other submission or publication in relation with this initiative.

### Quality assessment

Given the challenges in identifying a single assessment tool suitable for all papers included in this review, we adopted a mixed approach. The MMAT was used for research papers evaluating digital outcome measures, while the JBI critical appraisal tools were applied to narrative papers. As shown in Supplementary File S4, the quality of the narrative papers was excellent. All four papers satisfied all six criteria, indicating strong methodological rigor. For research papers, the overall quality was generally good; however, the most common weaknesses related to lack of clarity regarding population representativeness and missing outcome data.

## Discussion

Digital health is currently a major focus area, as attested by the increasing availability and use of wearable devices, the number of publications and the existence of dedicated journals. Digital outcomes from wearable devices are believed to have the potential to accelerate drug development, even if the formal demonstration has not been achieved yet. Regulatory authorities have expressed growing interest (28) in considering digital endpoints for drug approval. Beyond drug approval, wearable technology could also provide digital measures to assess disease progression in clinical practice and guide treatment strategy at the individual level (34). Devices are increasingly available, research on the topic is extensive, and dedicated journals exist. However, adoption remains limited. Understanding the reasons for this gap is essential to determine how it should be addressed.

First, our systematic review revealed that most studies were small-scale academic investigations, which often lacked robust datasets and a clearly articulated intention to pursue regulatory qualification. Nevertheless, they converge to demonstrate the feasibility of using wearable devices to assess medical conditions and the good compliance of patients, including in the pediatric population (35). However, even large initiatives such as Mobilise-D, a €50 million project funded by industry and the EU Innovative Medicines Initiative (IMI) project (36), faced major obstacles.

The Mobilise-D project has not yet delivered a validated functional digital mobility assessment tool or outcome (37). Still, it generated valuable insights and fostered collaboration with technology companies, clinicians, patient groups, and pharmaceutical companies. This collaborative model is a key strength. The focus on a wide range of medical conditions including Parkinson's disease, chronic obstructive pulmonary disease, MS and recovery from proximal femoral fracture, made challenging an impactful regulatory achievement in any of them. The structure of a major grant-funded project as a time-limited project further challenges the possibility to achieve regulatory qualification. Similarly, in MS and sarcopenia, early efforts including FDA Letters of Intent and algorithm validation studies have shown technical feasibility but failed to meet the stringent clinical and regulatory requirements for qualification. In Huntington disease, a 2020 LOI submission by MC10 was ultimately rejected, highlighting the difficulty of aligning device output with disease-specific regulatory standards. In contrast, the qualification of the SV95C for DMD by the EMA demonstrates that long-term, focused efforts, including multiple studies with strong metric properties and patient-level data, can result in regulatory success. However, this outcome remains confined to a narrow scope of application and cannot be directly extrapolated to other conditions without further validation.

Qualification is time- and resource-consuming. Optimizing project and study designs is essential. First, research efforts must align with regulatory expectations. This requires early and ongoing dialogue with agencies like the EMA and FDA. Second, long-term collaborations, between developers, clinicians, patient, and regulators is vital to ensure progress beyond the limits of individual projects or funding cycles. Third, studies should go beyond feasibility and compliance. They must rigorously evaluate the full psychometric properties of the outcome in target populations.

Another key strategy is to simplify the qualification process. Real-world gait assessment is relevant to many conditions—arthrosis, obesity, post-surgery, etc. Current regulatory frameworks require validation within a specific context of use. As a result, separate and extensive validation is needed for each medical condition. In contrast, the EMA provides a unified pathway for the approval of pain treatments across diseases (30) A similar regulatory approach could accelerate the use of digital outcomes. Bridging different context of use could support a faster implementation of digital outcomes across several indications. To promote generalizability, harmonized methodologies and transparent algorithms are also needed.

Beyond these primary steps, further challenges must be anticipated. Traditionally, regulatory evaluation of outcomes has been grounded in clinical meaning and patient relevance. For example, step count per day—measured via smartphone or other commercial devices—is clinically meaningful (38, 39) and easy to understand. However, daily variability limits its utility in clinical trials. Conversely, machine-learning-derived outcomes offer promises for prediction, early diagnosis, treatment selection, and personalized medicine. Yet, this metrics may lack transparency and perceived clinical relevance among patients and physicians. Moreover, it is crucial to focus on patient-centered digital health technologies to empower patients to manage and follow their activity. These should include intuitive apps and interfaces that present clear, relevant information. Easy interpretation by patients and clinicians will improve engagement, compliance, and overall clinical utility.

Together, these efforts could streamline qualification and promote the integration of digital endpoints in both regulatory processes and clinical practice. Recently, frameworks have been published to harmonize the development of patient-centered digital outcomes. All emphasize regulatory approval as a critical step toward integrating digital technologies into care (40, 41).

This systematic review has several limitations. First, the number of studies included in the systematic part of this review is small. This reflects the high specificity of our inclusion criteria and the limited volume of primary literature. To mitigate this, we complemented our systematic analysis with a narrative review. Second, we only reviewed the EMA and FDA websites, excluding other agencies such as PMDA in Japan or TGE in Australia. Lastly, we focused specifically on real-world walking speed, given its relevance across many conditions. A broader review of digital gait and mobility remains a future objective.

## Conclusion

Walking speed measured by wearable devices shows considerable promise in providing insightful data across a broad range of diseases and accelerate drug development. Yet, most published studies are insufficient to obtain regulatory approval, which is a critical step towards expanding digital outcome's reach in clinical practice. Accordingly, further research is necessary to comprehensively evaluate the clinical relevance of digital outcomes and achieve validation across a broader range of pathologies beyond DMD. Validation pathways must also be simplified and frameworks harmonized. It is of the utmost importance that studies are designed so that they can support the regulatory qualification of digital outcomes. With this approach, not only will they increase knowledge in the community, but they will also increase the number of tools that can directly find routine use.

## Data Availability

The original contributions presented in the study are included in the article/Supplementary Material, further inquiries can be directed to the corresponding author.
