# Supplementary material for: Real-world walking speed as a digital biomarker and outcome measure for clinical trials—a systematic review, regulatory status and future directions
Source: Front Digit Health. 2026 Feb 17;8:1726549. doi: 10.3389/fdgth.2026.1726549 (PMC12954611; doi:10.3389/fdgth.2026.1726549)
Supplement: Supplementary file 2 [file Table2.docx]

| Quantitative non-randomized controlled studies | | | | | |
| --- | --- | --- | --- | --- | --- |
| Reference | Are the participants representative of the target population? | Are measurements appropriate regarding both the outcome and intervention (or exposure)? | Are there complete outcome data? | Are the confounders accounted for in the design and analysis? | During the study period, is the intervention administered (or exposure occurred) as intended? |
| Mueller A., et al. 2019 | Y | Y | Y | Y | Y |
| Lilien C., et al. 2019 | Can’t tell | Y | Y | Y | Y |
| Poleur M., et al. 2021 | Y | Y | N | Y | Y |
| Gidaro T., et al. 2022 | Can’t tell | Y | N | Y | Y |
| Kirk, C. et al. 2023 | Y | Y | Y | Y | Y |
| Kirk, C. et al. 2024 | Y | Y | N | Y | Y |
| Buerkers J., et al. 2025 | Y | Y | Y | Y | Y |

| Narrative | | | | | | |
| --- | --- | --- | --- | --- | --- | --- |
| Reference | Is the generator of the narrative a credible or appropriate source? | Is the relationship between the text and its context explained? (where, when, who with, how) | Does the narrative present the events using a logical sequence so the reader or listener can understand how it unfolds? | Do you, as reader or listener of the narrative, arrive at similar conclusions to those drawn by the narrator? | Do the conclusions flow from the narrative account? | Do you consider this account to be a narrative? |
| Boehem P. et al. 2019 | Y | Y | Y | Y | Y | Y |
| Haberkamp M., et al. 2019. | Y | Y | Y | Y | Y | Y |
| Servais L. et al. 2021 | Y | Y | Y | Y | Y | Y |
| Servais L. et al. 2022 | Y | Y | Y | Y | Y | Y |
