# Supplementary material for: Real-world walking speed as a digital biomarker and outcome measure for clinical trials—a systematic review, regulatory status and future directions
Source: Front Digit Health. 2026 Feb 17;8:1726549. doi: 10.3389/fdgth.2026.1726549 (PMC12954611; doi:10.3389/fdgth.2026.1726549)
Supplement: Supplementary file 4 [file Datasheet1.pdf]

# SEARCH STRATEGY

## 1. EMBASE

#2 'mobile phone'
#3 'smartphone'
#4 'gyroscope sensor'
#5 'gyroscope'
#6 'accelerometer'
#7 wearable
#8 portable
#9 mobile
#10 digital
#11wireless
#12'inertial sensor'
#13 'devices'
#14 'tool'
#15 instrument
#16 'technology'#1 'accelerometry'/exp OR 'accelerometry'
#17 sensor
#18 biomarker
#19 #13 OR #14 OR #15 OR #16 OR #17 OR #18
#20 #7 OR #8 OR #9 OR #10 OR #11
#21 #19 AND #20
#22 #12 OR #21
#23 'real life'
#24 'remote sensing'
#25 'free living'
#26 'home'
#27 'real world'
#28 #23 OR #24 OR #25 OR #26 OR #27
#29 'velocity'
#30 walk
#31 'walking'
#32 'gait'
#33 'walking speed'
#34 #30 OR #31 OR #32
#35 #29 AND #34
#36 #33 OR #35
#37 #22 AND #28 AND #36
#38 #37 AND ('article'/it)
#39 #38 AND (2019:py OR 2020:py OR 2021:py OR 2022:py OR 2023:py OR 2024:py OR 2025:py OR 2026:py)

## 2. OVID/MEDLINE

- For device :
  1. accelerometry.mp. or exp Accelerometry/
  2. cell phone.ti, ab, kf.
  3. smartphone.ti, ab, kf.
  4. gyroscop.ti, ab, kf.
  5. accelero*.ti, ab, kf.
  6. Wearable.ti, ab, kf.
  7. portable.ti, ab, kf.
  8. mobile.ti, ab, kf.
  9. digital .ti, ab, kf.
  10. wireless.ti, ab, kf.

11. inertia*.ti, ab, kf.
12. 6 OR 7 OR 8 OR 9 OR 10 OR 11
13. device.ti, ab, kf.
14. tool.ti, ab, kf.
15. Instrument*.ti, ab, kf.
16. technolog*.ti, ab, kf.
17. Sensor*.ti, ab, kf.
18. biomarker.ti, ab, kf.
19. phone.ti, ab, kf.
20. 13 OR 14 OR 15 OR 16 OR 17 OR 18 OR 19
21. 12 AND 20
22. 1 OR 2 OR 3 OR 4 OR 5 OR 21

- For real-life:
  23. Real-life.ti, ab, kf.
  24. Remote.ti, ab, kf.
  25. Free-living.ti, ab, kf
  26. home.ti, ab, kf
  27. Real-world.ti, ab, kf
  28. 23 OR 24 OR 25 OR 26 OR 27
- For outcome:
  29. Speed.ti, ab, kf
  30. Velocity.ti, ab, kf
  31. walk*.ti, ab, kf.
  32. Gait.ti, ab, kf.
  33. Stride.ti, ab, kf.
  34. Step*.ti, ab, kf.
  35. 29 OR 30
  36. 31 OR 32 OR 33 OR 34
  37. 35 AND 36
- Research
  38. 22 AND 28 AND 37
- Limits:
  Limit to yr="2019 -Current”
  Limit to english
